# Therapeutic cancer prevention: achievements and ongoing challenges – a focus on breast and colorectal cancer

**DOI:** 10.1002/1878-0261.12461

**Published:** 2019-02-21

**Authors:** Davide Serrano, Bernardo Bonanni, Karen Brown

**Affiliations:** ^1^ Division of Cancer Prevention and Genetics European Institute of Oncology IRCCS Milan Italy; ^2^ Leicester Cancer Research Centre University of Leicester UK

**Keywords:** aspirin, breast cancer, colorectal cancer, medical prevention, tamoxifen, therapeutic prevention

## Abstract

The constant increase of cancer incidence and the huge costs of new treatments make cancer prevention a crucial goal in order to maintain sustainable public health systems across the world. Carcinogenesis is a multistep process, which allows time for active intervention with natural or synthetic agents to stop or reverse the pathological process. Cancer prevention medicine can be considered to be treatment of premalignant cells or preneoplastic conditions. Clearly such interventions require well‐defined risk classification so that personalized strategies and specific treatments can be applied to cohorts with a documented increased cancer risk, and not to the general population as a whole. Further development of these strategies in an efficient and timely manner requires investment in the discovery and validation of surrogate cancer biomarkers with both prognostic and predictive value to detect and monitor the efficacy of interventions in clinical trials and beyond. In the field of cancer prevention medicine, breast and colon cancer demonstrates the strongest clinical evidence that pharmacological intervention can lower cancer risk. Here, we offer an overview of the major clinical achievements for these cancers and the critical issues to improve implementation and clinical uptake of efficacious therapies, as well as further developments needed in the field of preventive medicine.

**Abbreviations**
AIs: aromatase inhibitors
ASCO: American Society of Clinical Oncology
BCS: breast conservation surgery
CAPP: colorectal adenoma/carcinoma prevention programme
CI: confidence intervals
CRC: colorectal cancer
CVD: cardiovascular disease
DCIS: ductal carcinoma *in situ*
ER: estrogen receptor
FAP: familial adenomatous polyposis
FDA: US Food and Drug Administration
HR: hazard ratio
IBIS‐II: International Breast Cancer Intervention Study II
IEN: IntraEpithelial Neoplasia
LCIS: lobular carcinoma *in situ*
LDL: low‐density cholesterol
NICE: National Institute for Health and Care Excellence
NSABP: National Surgical Adjuvant Breast and Bowel Project
NSAID: nonsteroidal anti‐inflammatory drug
OR: odds ratio
PreSAP: prevention of colorectal sporadic adenomatous polyps
RR: relative risk
RT: radiation therapy
SERMs: selective estrogen receptor modulators
UK/ANZ DCIS: United Kingdom, Australia, and New Zealand ductal carcinoma *in situ*

## 1 Introduction

### 1.1 The need for cancer prevention

The cancer burden and death from cancer are increasing worldwide. More than 14 million cases occur every year, but this number is estimated to reach nearly 22 million globally by 2030 (Bray *et al*., 2015). The escalating costs of diagnosing and managing cancer are clearly not sustainable for public health structures, particularly considering the high price of newer cancer treatments. Importantly, the problem is not restricted to high‐income countries, the incidence of cancer is also increasing in low‐ to middle‐income countries where is it projected to account for ~60% of the world total by 2030 (Bray and Soerjomataram, 2015). Geographical diversity is still relatively evident for site‐specific cancer incidence (e.g., cervical cancer incidence rates are high in sub‐Saharan regions and some Latin American countries), but a more ‘globalized’ cancer burden has shown a rapid increase in malignancies typically associated with a Western lifestyle, such as lung, colon, and breast cancers, worldwide (Bray *et al*., 2015). There is an urgent need to change this scenario, and one of the most realistic routes is by boosting early detection and prevention strategies.

Early detection has played a significant role in reducing the economic burden of cancer, due to the lower treatment costs, and morbidity and mortality of cancers when diagnosed at an early, compared to advanced, stage. Preventive interventions, including lifestyle and behavioral changes and use of preventive medicine (or therapies), still need far more effort in terms of development and implementation, from basic research up to the educational and communication level, to realize their true potential. Preventive medicines, by virtue of the fact they are normally given to healthy people who do not have cancer, must be safe and well tolerated; this restricts the choice of candidate therapies to vaccines, repurposed, established (typically generic) drugs, and certain dietary‐derived compounds, where there is good evidence of safety. All these options are much more affordable compared to standard health care for a cancer patient, especially when including the newest target therapy. Moreover, avoiding the strong psychological impact of a cancer diagnosis for individuals and their family is invaluable.

### 1.2 Cardiovascular disease has led the way

Therapeutic prevention is standard practice in cardiovascular disease (CVD) where use of antihypertensives, statins and antiplatelet drugs have contributed to a dramatic decline in mortality due to CVD over the past ~40 years (Hansson, 2005) The success in CVD prevention can be attributed to the relatively straight forward relationship between the disease and related biomarkers. For example, high blood pressure or high low‐density cholesterol (LDL) can be considered disease surrogate biomarkers, they are easily monitored, their modulation by diet or treatment can be clearly quantified, and both the subjects and physicians can perceive the benefits of intervention (Zethelius *et al*., 2008) (Brown and O'Connor, 2010; Jemal *et al*., 2010).

### 1.3 Challenges in therapeutic prevention

**Figure 1:**
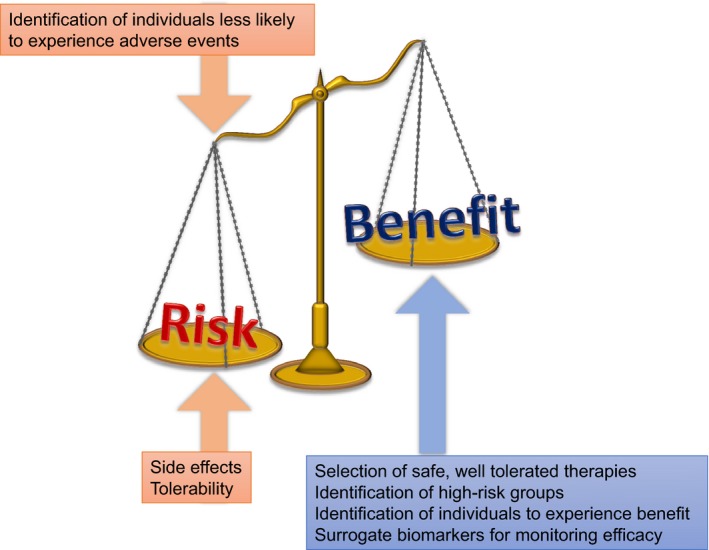
Factors that influence the risk:benefit ratio of preventive therapies. Better identification of individuals at increased risk, together with those most likely to experience a net benefit from a specific intervention will favorably alter the risk:benefit ratio. Improved availability of validated surrogate cancer biomarkers will allow quicker assessment of efficacy in clinical trials and continuous monitoring of potential efficacy once therapies are more widely used. Progress in these areas must be underpinned by a greater mechanistic understanding of cancer risk factors and modes of action for each preventive therapy.

The development of cancer prevention medicine is hampered by the lack of established and validated surrogate biomarkers, and all strategies aiming to lower cancer incidence, from lifestyle to therapeutic interventions, are challenging to develop and implement since subjects and physicians have no means of measuring whether the interventions might be effective in the short‐medium term. Effects on cancer occurrence, as the definite endpoint, can take decades to evaluate. In order to reach standard clinical practice, similar to the situation in CVD, cancer prevention medicine will require a major effort to discover and validate surrogate biomarkers that will allow better identification of individuals at risk, as well as monitoring the efficacy of interventions within clinical trials (Fig. 1). A greater understanding of modes of action and mechanisms of toxicity for therapies under investigation will also enable identification of those individuals most likely to benefit, and those at risk of experiencing side effects, which all contribute to a more favorable risk–benefit ratio. For breast cancer, mammographic breast density might be a realistic surrogate marker, as it predicts future cancer risk and is modifiable by interventions over a relatively short interval; reductions in breast density have been shown to be an excellent predictor of response to tamoxifen in the prevention setting (Cuzick *et al*., 2011c). As precursors to most colorectal cancers, adenomatous polyps are the only validated surrogate endpoint for this cancer, but trials assessing preventive efficacy against sporadic adenomas typically last for at least a year and require large number of patients (Hull *et al*., 2013) (Baron *et al*., 2003). Consequently, new short‐term biomarkers are urgently needed for this cancer and the same deficiencies are evident, or even worse, across the majority of other malignancies that also require prevention strategies.

Another big issue that needs improving is communication between physicians and potential candidates for preventive therapy, to estimate, calculate, and explain their risks, and illustrate the possible benefits of the options available.

In this article, we highlight the major achievements in therapeutic prevention, which are best illustrated by the progress made in breast and colorectal cancer. We also address the barriers that need to be overcome in order to move this promising field forward and bring about the reductions in cancer incidence that are desperately needed.

## 2 The scope of therapeutic/medical cancer prevention

The concept of ‘chemoprevention’ as an approach to reducing cancer incidence that considers the whole disease process, not just the final invasive manifestation, was first introduced by Michael Sporn in 1976 (Sporn, 1976). It can be defined as the use of natural, synthetic, or biological agents to reverse, suppress, delay, or prevent either the initial phases of carcinogenesis or the progression of premalignant cells to invasive disease (Steward and Brown, 2013). Interestingly, it has been recognized that use of the term ‘chemoprevention’ can evoke inappropriate associations with cancer and chemotherapy, which has a negative impact on uptake by eligible individuals; therefore, alternatives such as therapeutic or medical prevention are advocated (Cuzick *et al*., 2011a). For successful implementation of specific preventive therapies or combinations, it is crucial to identify an appropriate cohort at risk of cancer and a possible target phenotype, such as hormonal or metabolic imbalance, or the presence of subclinical inflammation. Advances in basic science will lead to new hypotheses regarding risk factors, and once validated, results can be used to refine existing and develop new risk models. Areas of interest include gene alterations (from polymorphisms to deleterious mutations with different penetrance and cancer risk (Foulkes, 2008)), metabolic factors and microbiota; taken together a better understanding of these factors and others will help define the balance between inner predisposition and susceptibility to environmental exposures (Hursting *et al*., 2012).

Effective preventive medicine strategies already exist, but they are not yet being fully implemented, for a number of reasons: physicians’ lack of knowledge about cancer risk evaluation tools and preventive therapy; patients’ level of awareness of their risk, leading to underestimate the role of a preventive treatment, or on the other hand overestimate the potential harms (Decensi *et al*., 2015). Certainly, it is clear that more high‐quality information and training on evidence‐based prevention strategies have to be delivered to all relevant healthcare professionals and that appropriate counseling needs to be more available to potential patients on the possible effective preventive therapies they could take, in the context of their own specific risk (Waters *et al*., 2010).

**Figure 2:**
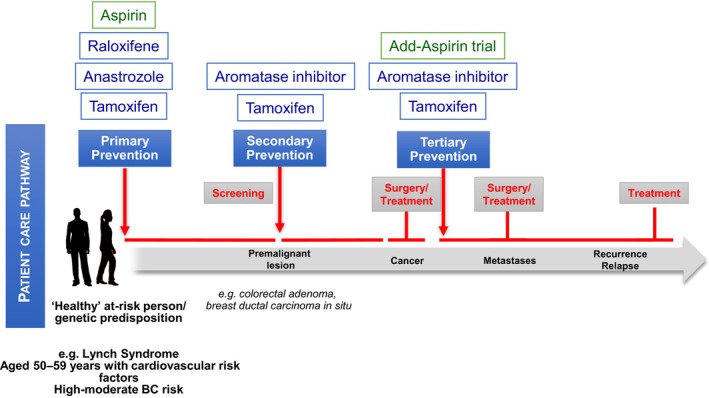
Cancer development and prevention opportunities. Therapeutic interventions can generally be implemented at three different stages of cancer development, resulting in primary, secondary, or tertiary prevention paradigms. This figure illustrates how prevention opportunities map onto the patient care pathway for colorectal and breast cancer. Also shown are drugs that are currently used or being investigated in trials for the different types of prevention.

Therapeutic prevention can be described as primary, secondary, or tertiary, according to the population being targeted and stage of cancer development (Fig. 2). The aim of primary prevention in a broader sense is to maintain a healthy condition in asymptomatic subjects and so it also encompasses avoiding or minimizing exposure to known carcinogens or other agents that might contribute to carcinogenesis (e.g., tobacco smoking, alcohol consumption, red or processed meat), as well as implementing beneficial habits (e.g., physical activity and diet rich in vegetables and fruit) through education and behavioral change. Classic primary prevention therefore equates to the introduction of practices and policies that cover the general population, such as banning of smoking in public places or implementation of healthier diets in the school/work place cafeteria. In individuals with a higher than average cancer risk, for example due to an inherited predisposition, then additional therapeutic prevention approaches are warranted in addition to any general recommendations. These include the use of selective estrogen receptor modulators (SERMs) in healthy women at high risk of developing breast cancer due to a family history and other personal risk factors, or aspirin for colorectal cancer prevention in individuals with Lynch syndrome, or people aged 50–59 years, who are also at high risk for cardiovascular disease. For the highest risk cohorts of the germline mutation carriers (e.g., mutations of APC, BRCA1, BRCA2, or CDH1), cancer prevention can ultimately extend to prophylactic surgery.

Among secondary prevention, therapeutic prevention addresses subjects with intermediate risk conditions or cancer precursors such as intraepithelial neoplasia (from atypical hyperplasia to intraductal carcinoma for the breast) or colorectal adenomas. Moreover, due to improved survival, increasing numbers of patients that have been successfully treated for cancer require tertiary prevention, which again can span from lifestyle modification to therapeutic interventions such as SERMs and aromatase inhibitors (AIs).

Overall tertiary prevention aims to reduce morbidity and disability for an ongoing disease, and more specifically in cancer patients, the main goal is to prevent second primary malignancies, or other long‐term treatment‐related complications. Tertiary prevention is currently becoming very relevant due to the growing number of cancer survivors.

## 3 Achievements in breast cancer prevention

Tamoxifen has been routinely used since the 1980s for the adjuvant treatment of breast cancer to reduce the risk of both recurrence and developing invasive disease; this indication equates to tertiary prevention (Fig. 2). In 1998, the US Food and Drug Administration (FDA) approved the use of tamoxifen for breast cancer risk reduction in both pre‐ and postmenopausal women with an increased risk, based on the Gail model (Gail and Benichou, 1994). Subsequently, the FDA also approved raloxifene for primary breast cancer prevention in postmenopausal women. Both the ASCO and UK National Institute for Health and Care Excellence (NICE) guidelines suggest discussions should be had with women at moderate‐high risk regarding the use of preventive treatment with SERMs or AIs (National institute for health and clinical excellence, 2013; Visvanathan *et al*., 2013).

### 3.1 Primary prevention of breast cancer

**Figure 3:**
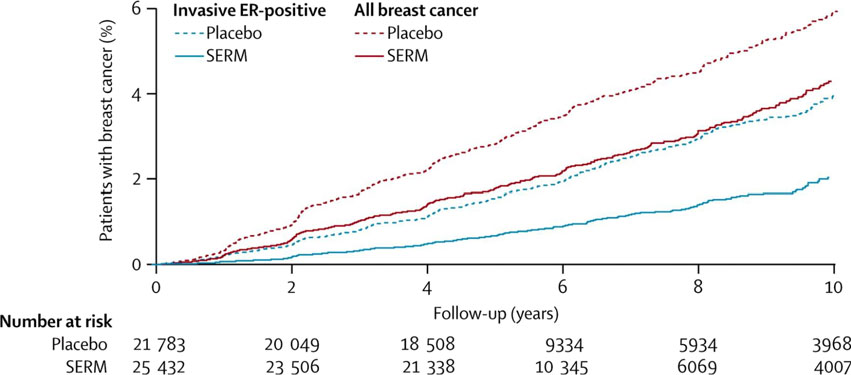
Cumulative incidence for all breast cancer (including ductal carcinoma *in situ*) and all ER‐positive invasive cancers in years 0–10 according to treatment allocation. SERM, selective estrogen receptor modulator; ER, estrogen receptor. Figure reproduced with permission from Cuzick et al (2013).

The benefits of primary prevention are well documented in the long‐term SERMs meta‐analysis by Cuzick *et al*. (Cuzick *et al*., 2013) (Fig. 3). This meta‐analysis showed a statistically significant overall reduction in all breast cancers of 38% and indicated that 42 women would need to be treated to prevent one breast cancer in the first ten years of follow‐up. The analysis included four tamoxifen prevention trials involving more than 28 000 subjects and a median follow‐up of 116 months; three raloxifene studies with more than 37 000 women with a median follow‐up of 73 months; and two other studies with newer SERMs (lasofoxifene and arzoxifene). Tamoxifen risk reduction was more pronounced in the first 5 years but was still significant in years 5–10 of follow‐up. Compared to placebo, tamoxifen showed a reduction of 33% for all breast cancers; this effect was restricted to ER‐positive invasive disease where the reduction reached 44%. In contrast, a nonsignificant increase in estrogen receptor negative breast cancers was observed. Tamoxifen use was also associated with a significant 31% reduction in cases of ductal carcinoma *in situ* (DCIS), which is characterized by clonal proliferation of epithelial cells confined within the lumen of mammary ducts. This benefit was only evident with tamoxifen, as the other SERMs did not reduce DCIS. The two major adverse events caused by tamoxifen, endometrial cancer and venous thrombosis, were further confirmed in the meta‐analysis. The rate of endometrial cancer was significantly increased in those women taking SERMs (HR 1.64, 1.14–2.36; *P* = 0.007); however, this effect was confined to the tamoxifen trials and was limited to the first 5 years; it was not apparent during years 5–10 after treatment had ceased. Venous thromboembolic events were significantly increased by all SERMs (OR 1.60, 95% CI 1.21–2.12 and 1.45, 95% CI 1.18–1.76, respectively, for tamoxifen and raloxifene), but again the difference was only while women were on treatment. No differences due to SERM use were seen for other cardiovascular disorders.

### 3.2 Barriers to implementation

Even though the long‐term follow‐up data reinforce the initial beneficial findings from all the prevention trials, the routine use of primary prevention is still very low, which is currently limiting its effectiveness at a population level (Smith *et al*., 2016). It has been estimated that ~2 million US women and 0.5 million women in the UK meet the eligibility requirement for tamoxifen prophylactic therapy but only one in six accept the offer, with significantly lower rates in nontrial settings (Smith *et al*., 2016) (Smith *et al*., 2017). Contributing factors to low uptake include concerns over side effects, a lack of awareness by potential prescribers that tamoxifen could reduce the risk of breast cancer and the existence of guidelines advocating its use in this setting, and the requirement to prescribe off‐label in countries where tamoxifen is not licensed for prevention (Smith *et al*., 2017),(Smith *et al*., 2016). In April 2018, twenty years after it was approved by the FDA, the UK Medicines and Healthcare products Regulatory Agency approved the indication for tamoxifen in the primary prevention of breast cancer in women at moderate or high risk. It will be interesting to examine whether this approval alters the behavior of potential prescribers and improves the wider implementation of tamoxifen. More generally, improvements in uptake may also come through greater awareness and offering of aromatase inhibitors in the future, as these have improved efficacy while lacking the endometrial cancer and thrombogenic risks associated with tamoxifen.

### 3.3 Secondary prevention in women with intraepithelial neoplasia

A different scenario can be considered for women who have already received a diagnosis of breast IntraEpithelial Neoplasia (IEN), as these individuals might be more aware of their risk, which should make preventive medicine more sustainable over the long periods required for protection. Tamoxifen has already shown a positive effect both for intraductal and for intralobular neoplasia.

Two main randomized phase III trials have documented the validity of tamoxifen use in women with DCIS after complete local excision, lowering the risk for a second ipsilateral or contralateral new event by approximately 30%, both at 10 and at 15 years (Cuzick *et al*., 2011b; Wapnir *et al*., 2011). The NSABP B24 trial showed a 32% reduction in invasive ipsilateral recurrences and contralateral breast cancers with tamoxifen use. A reduction in DCIS ipsilateral recurrences was noted but failed to reach statistical significance (Wapnir *et al*., 2011). Another trial, the UK/ANZ DCIS study, did not show a statistically significant benefit of adding tamoxifen treatment at an initial analysis after a median follow‐up of 4.4 years. However, a subsequent updated analysis after a longer median follow‐up of 12.7 years, when 376 breast cancers had been diagnosed, revealed that tamoxifen reduced the incidence of all new breast events (HR 0.71, 95% CI 0.58–0.88), reducing recurrent ipsilateral DCIS (0.70, 0.51–0.86) and contralateral tumors (0.44, 0.25–0.77), but having no effect on ipsilateral invasive disease (Cuzick *et al*., 2011b).

More recently, aromatase inhibitors have been compared to tamoxifen in two phase III trials for secondary prevention (Forbes *et al*., 2016; Margolese *et al*., 2016). IBIS‐II (DCIS), a double‐blind randomized placebo‐controlled trial, involved 2980 postmenopausal patients with hormone receptor‐positive DCIS, treated with conservative breast surgery (BCS) with or without RT. Subjects were randomly assigned to anastrozole 1 mg·day^−1^ or tamoxifen 20 mg·day^−1^ for 5 years. No statistically significant difference in overall recurrence was observed between the arms (median follow‐up of 7.2 years). The side effect profiles of the two drugs were different, but the overall number were similar in the two arms (Forbes *et al*., 2016). The NSABP B‐35 trial randomized 3104 postmenopausal women with DCIS to tamoxifen or anastrozole. No clear differences were seen overall, but for younger women (<60 years) anastrozole was more effective than tamoxifen. Anastrozole was also associated with a significant 36% reduction in total contralateral events. Similar to the IBSI II study, the toxicity of the two drugs differed but there was no real evidence of superiority of one agent over the other. These differences may, however, allow personalization of treatment based on patient characteristics (Margolese *et al*., 2016).

Women with atypical hyperplasia or lobular carcinoma *in situ* (LCIS) have at least a ~4‐fold increased risk of breast cancer compared to the general population and may also gain advantage from the use of endocrine preventive therapy to reduce a second mammary event (Coopey *et al*., 2012). A recent observational study in over a thousand women, after a diagnosis of lobular carcinoma *in situ*, showed a significant breast cancer risk reduction (HR, 0.27; 95% CI 0.15 to 0.50). A second study, in subjects with previous atypical hyperplasia, including LCIS, also demonstrated considerable tamoxifen risk reduction with overall decreases of 52% for invasive cancers and 55% for *in situ* neoplasia (Coopey *et al*., 2012; King *et al*., 2015).

The standard dose of tamoxifen used in prevention trials is 20 mg daily, but lower doses (5 mg per day or 10 mg every other day) have also been show to offer advantages in women treated for an estrogen receptor‐positive ductal neoplasia. Analysis of an observational cohort revealed that low‐dose endocrine treatment decreased any breast event (HR 0.70, 95% CI: 0.54–0.91) and ipsilateral DCIS recurrence (HR 0.66, 95% CI: 0.49–0.88), and it was more effective on all breast events in women aged >50 years. The study included 1091 women, median follow‐up of 7.7 years, with a 235 ipsilateral recurrences and 62 contralateral breast tumors (Guerrieri‐Gonzaga *et al*., 2016). A phase III randomized trial in IEN patients with low‐dose tamoxifen is ongoing (Zanardi *et al*., 2011).

It is important that all women at moderate‐high risk for breast cancer receive the information that an effective possibility to lower the risk exists. It is crucial to develop strategies to sensitize healthcare providers and women to discuss this opportunity. It is important that women can make their own decision, after being provided a balanced information on the potential treatment benefits and risks. For example, tamoxifen has a very well‐known side effects profile, and based on the subject's health history and lifestyle, it is possible to make an estimate of the individual risk/benefit ratio. In postmenopausal women, if tamoxifen cannot be advisable, aromatase inhibitors can be considered as an alternative (Cuzick *et al*., 2014; Goss *et al*., 2011). Furthermore, tamoxifen at lower doses is becoming a clinical opportunity (A. DeCensi oral presentation SABCS 2018).

## 4 Colon cancer: from sporadic cancer to hereditary syndromes

Colorectal cancer (CRC) is the third most common cancer and the fourth leading cause of cancer‐related deaths worldwide (Ferlay *et al*., 2010). Aspirin and nonsteroidal anti‐inflammatory drug (NSAID) use has been associated with reduced risk of CRC in several studies, initially with contradictory results but longer follow‐up subsequently confirmed the positive effects. A meta‐analysis of epidemiological studies, including a total of 16 105 cases, showed a 27% risk reduction of CRC with aspirin use (Bosetti *et al*., 2012). The protection was already evident within the first 5 years and increased with longer duration of use (RR 0.80 95% CI 0.71–0.91 < 5 years and 0.75 (95% CI 0.70–0.80) for ≥5 years).

Rothwell *et al*. conducted a meta‐analysis on eight randomized trials of aspirin for cardiovascular prevention. Daily aspirin treatment, using doses between 75 and 1200 mg per day, reduced deaths due to several common cancers, with the benefit being apparent only after 5 years of follow‐up. There was a nonsignificant 21% reduction in colorectal cancer up to 10 years, which became a significant 49% decrease between 10 and 20 years and remained significant (40% reduction) with longer follow‐up (Rothwell *et al*., 2011). More specifically, for colorectal cancer the same author showed that a minimum of 75 mg daily aspirin taken for several years reduced not only mortality but also incidence of colorectal cancer. Separate evaluation of data on cancers of the colon and rectum revealed that incidence was significantly reduced for colon cancer (24% risk reduction *P* = 0.02) but not for rectal cancer (HR 0.90, 0.63–1.30, *P* = 0.58) (Rothwell *et al*., 2010). Furthermore, studies where the data on proximal and distal colon were available suggest a more pronounced effect on the proximal colon (HR 0.45, 0.28–0.74, *P* = 0.001). However, with a longer follow‐up a significant reduction in incidence was reached in all colorectal segments (Rothwell *et al*., 2010). A recent meta‐analysis on epidemiological studies addressed dose–risk and duration–risk relationships. It confirmed that long term (at least 5 years), low dose (75–325 mg per day), and regular aspirin use (2–7 times per week) can effectively reduce colorectal cancer risk (Ye *et al*., 2013).

As illustrated in Fig. 2, colorectal cancer prevention can be addressed to subjects with a positive family history for colorectal cancer (primary), a personal history of adenoma (secondary), or to participants with an early‐stage colorectal cancer where no adjuvant treatment is considered, as is currently being investigated in the ongoing Add‐Aspirin trial (tertiary http://www.addaspirintrial.org/). Two large randomized trials have shown that aspirin reduces colorectal adenomas. Baron *et al*. (2003) randomized 1121 subjects with a history of adenomas. The study arms were aspirin 325 mg, or 81 mg daily or placebo. The results revealed a statistically significant reduction in adenoma recurrence in the aspirin groups versus placebo (*P *=* *0.04). Per arm, the relative risk was 0.81 (95% CI 0.69–0.96) in the 81 mg and 0.96 (95% CI 0.81–1.13) in the 325 mg aspirin arm. Interestingly, the effect was stronger for the lower dose and for advanced lesions compared to tubular adenoma.

The second adenoma study by Sandler *et al*. (2003) included 635 CRC patients (Dukes’ stage A, B1 soon after surgery or B2, C after 5 years disease‐free survival). Patients were randomized to receive aspirin 325 mg daily or placebo. In the aspirin arm, 17% of patients developed at least one adenoma vs. 27% in the placebo arm (*P *=* *0.004, RR 0.65 95% CI 0.46–0.91). Interesting observations in this study were that aspirin delayed the time of adenoma insurgence, and there was no significant difference in toxicity between the two arms. Overall the drug tolerability was acceptable in both trials, except for a borderline significant (*P *=* *0.06) increased incidence of stroke in the aspirin group of the Baron study.

As a result of the current clinical evidence, aspirin is gaining acceptance as a colorectal cancer preventive therapy in age‐stratified groups, as highlighted by the recently updated US Preventive Services Task Force guidelines which recommend aspirin for primary cancer prevention in individuals aged 50–59 years, who also have a 10% or greater 10‐year risk of cardiovascular disease (Bibbins‐Domingo, 2016). A risk–benefit analysis has calculated that aspirin could actually prevent more deaths due to cancer than cardiovascular disease in the general population and proposed that prophylactic use of aspirin (75–325 mg·day^−1^) for a minimum of 5 years in the age range 55–65 would have a favorable risk–benefit ratio and a net 4% relative reduction in all deaths (Cuzick *et al*., 2015).

Similar to aspirin, COX‐2 inhibitors have also been reported to lower adenoma recurrence in subjects with previous adenoma history. The PreSAP study randomized 1561 subjects to 400 mg celecoxib or placebo. The results clearly confirm that at 5‐year follow‐up there was significantly lower adenoma recurrence in the celecoxib group (RR 0.64; 0.56–0.75 95% CI; *P* < 0.001) (Arber *et al*., 2006). In another trial from the Bertagnolli group, 2035 subjects were allocated either placebo, celecoxib 200 mg twice daily, or celecoxib 400 mg twice daily. At 5 years of follow‐up, a significant 30–40% reduction in recurrence was detected (RR 0.67; 0.59–0.77 95% CI and 0.55; 0.48–0.64 95% CI for 200 mg twice daily, and 400 mg twice daily, respectively) (Bertagnolli *et al*., 2006). Both studies confirmed the reduction of colorectal adenoma formation due to celecoxib, but also an increased risk for cardiovascular and thrombotic events. Serious adverse events reported by the Bertagnolli group had a RR, of 1.1 (95% CI, 0.9–1.3; *P* = 0.5) and 1.2 (95% CI 1.0–1.5; *P* = 0.06), respectively, for the low‐ and high‐dose group versus placebo. The conclusion was that celecoxib should not be routinely recommended (Bertagnolli *et al*., 2006).

If NSAIDs, and in particular aspirin, can be recommended to higher risk subjects for sporadic cancer, what about hereditary syndromes? Giardiello *et al*. (1993) showed that nine months of sulindac reduced the number of adenomas by 44% in patients with familial adenomatous polyposis (FAP). Then, a small, but significant, study led the FDA to approve celecoxib as preventive agent in this syndrome. In fact, six months of celecoxib 400 mg twice a day showed a 28% reduction in the mean number of colorectal polyps in young adults (Steinbach *et al*., 2000).

The CAPP2 study was the first large‐scale chemoprevention trial with aspirin focused on Lynch syndrome. A total of 861 participants were randomly assigned to aspirin or placebo. A first publication (Burn *et al*., 2008) did not show significant results, but with a longer follow‐up aspirin efficacy became evident. Overall, the intention‐to‐treat analysis showed a nonsignificant 37% cancer reduction; however, the effect became significant when patients with multiple primary colorectal cancers were included and the reduction reached 44% (*P* = 0.05). Furthermore, when considering compliant subjects (with at least two years of aspirin use), the reduction was 59% (*P* = 0.02). If we consider not only colorectal but all the syndrome‐related cancers, a significant 45% cancer reduction (*P* = 0.05) was estimated at the intention‐to‐treat analysis (Burn *et al*., 2011). A current trial, CAPP3, is ongoing and will address the best dose and duration of aspirin treatment in Lynch syndrome carriers (Burn *et al*., 2013).

## 5 Promising agents under investigation

Among the endless list of agents with cancer preventive potential, we mention here only two of them, metformin and vitamin D since, to our knowledge they have accumulated sufficient supporting evidence to encourage the design of phase III trials (www.clinicaltrial.gov).

### 5.1 Metformin

For the antidiabetic drug metformin, prevention of carcinogenesis is at least partly through its systemic insulin‐lowering activity, which decreases cell proliferation in individuals with hyperinsulinemia, together with its ability to induce autophagy in preneoplastic and neoplastic cells via direct effects on pathways such as AMPK and mTOR signaling (Han *et al*., 2015; Pollak, 2012). Metformin is associated with reduced overall cancer incidence and mortality in observational studies. In a meta‐analysis of 12 studies, a borderline significant risk reduction for colon cancer was shown, with a summary relative risk (SRR) 0.80, 95% CI, 0.64–1.00 (Gandini *et al*., 2014). Evidence is similar for colorectal adenoma, with metformin use associated with a significant 24% reduction in adenoma recurrence (Jung *et al*., 2017). These data are supported by a double‐blind, placebo‐controlled trial of low‐dose metformin (250 mg·day^−1^) in high‐risk nondiabetic patients that had previously had polyps/adenomas endoscopically resected. After intervention for one year, metformin decreased adenoma recurrence by 40% compared to placebo (Higurashi *et al*., 2016). This particular trial was conducted in Japan, and further studies are warranted to confirm efficacy and ascertain whether the protective effects translate to Western populations.

Metformin has a good safety profile and usually it is well tolerated by patients. The side effects that may require discontinuation are gastrointestinal, mainly diarrhea which is usually self‐limiting. The only potential major adverse event in metformin therapy is lactic acidosis, but this condition is fortunately very rare.

### 5.2 Vitamin D

Calcitriol, the active metabolite of vitamin D, is involved in multiple signaling pathways that can regulate cell proliferation, apoptosis, differentiation, inflammation, invasion, angiogenesis and metastasis. Ample *in vitro* evidence has demonstrated the potential for vitamin D to affect cancer development and growth (Feldman *et al*., 2014). Epidemiological and preclinical studies support the role of vitamin D as a preventive agent, with low vitamin D status being significantly associated with overall mortality and cancer outcome, more than cancer incidence (Tagliabue *et al*., 2015). A meta‐analysis has shown a significant inverse relationship between 25‐hydroxyvitamin D levels and colorectal cancer risk with a SRR 0.85 (95% CI 0.79–0.91, *P* = 0.004) (Gandini *et al*., 2011). In another meta‐analysis, vitamin D levels showed inverse association with colorectal cancer (OR 0.66; 95% CI, 0.54–0.81), with a stronger reduced risk for rectal cancer (OR 0.50; 95% CI, 0.28–0.88) (Lee *et al*., 2011).

## 6 Conclusions

Clinical evidence has convincingly demonstrated that it is possible to lower cancer incidence, at least for breast and colorectal cancer, through the use of preventive medicine. Despite these data many physicians have displayed a reluctance to suggest this option to eligible individuals. In addition, subjects who could take advantage of cancer preventive medicine have low acceptance rates and poor adherence to such program. Several reasons may be involved, but probably the most significant is the lack of surrogate endpoint biomarkers. The success of cardiovascular prevention can be attributed to the simple and accessible surrogate biomarkers, such as high blood pressure or cholesterol, since they are easy to monitor and provide a concrete readout of treatment efficacy. The lack of an objective marker to target with preventive cancer therapies makes it more difficult for physicians to advise, and for candidates to undergo, treatment without a measurable outcome, particularly as they may not themselves experience a benefit but could suffer side effects.

In this field, risk perception, information and counseling are crucial. The discussion between healthcare professionals and the person eligible for preventive therapy has to equip the individual with information, presented in an appropriate manner, that allows them to decide by him/herself whether the treatment is a reasonable option, in light of personal preferences and values. The aim of cancer prevention is to avoid the diagnosis of cancer with all the physical, psychological, and social implications it brings. The latter aim is the primary goal of screening programs and adjuvant trials where the purpose is to improve early detection and micro‐metastases control/eradication respectively, and therefore mortality.

Population selection is another crucial issue for a pharmacological intervention. This aspect was particularly evident in the Italian tamoxifen study where the average breast cancer risk of the participants was medium/low and the breast cancer reduction in those receiving tamoxifen was not statistically significant, whereas, in the subgroup analysis of higher risk subjects, tamoxifen was extremely effective, consistent with the other tamoxifen chemoprevention trials (Veronesi *et al*., 2007).

Beside anamnestic information, several tools (computer‐assisted algorithms) are now available to evaluate personal cancer risk. Based on the risk level, all the different options to antagonize the risk should be discussed, from lifestyle, to pharmacological interventions and prophylactic surgery in hereditary cancer syndromes.

Implementing awareness and education on cancer prevention is mandatory for both the general public and healthcare professionals. Presenting objectively the different options, after a careful evaluation of the individual risk level, should be considered good clinical practice by every physician, balancing the efficacy and long‐term potential for benefit, even after treatment discontinuation (see the long‐term efficacy of tamoxifen (Cuzick *et al*., 2013)) with the potential risks and adverse effects of chemoprevention agents.

## Author contributions

DS was involved in manuscript preparation, BB manuscript revision and KB manuscript preparation and revision.

## Conflict of interest

The authors declare no conflict of interest.
